# Validity and reliability of center of pressure measures to quantify trunk control ability in individuals after stroke in subacute phase during unstable sitting test

**DOI:** 10.1016/j.heliyon.2022.e10891

**Published:** 2022-10-03

**Authors:** Anne-Violette Bruyneel, Serge Mesure, Aline Reinmann, Caroline Sordet, Pablo Venturelli, Irmgard Feldmann, Emmanuel Guyen

**Affiliations:** a Geneva School of Health Sciences, HES-SO University of Applied Sciences and Arts Western, Switzerland; b Institute of Movement Sciences, National Center of Scientific Research, Aix-Marseille University, Marseille, France; c Division of Neurorehabilitation, Department of Clinical Neurosciences, University Hospital of Geneva, Geneva, 1211, Switzerland

**Keywords:** Stroke, Validity, Reliability, Sitting position, Posture

## Abstract

**Objective:**

The objective of this study was to assess, for individuals with hemiparesis after a stroke in subacute phase, the validity and reliability of center of pressure (CoP) parameters measured during sitting balance on an unstable support.

**Materials and methods:**

Thirty-two individuals after stroke were included in this observational study for validity and reliability (mean age: 64.34 ± 9.30y, 23 men, mean post-stroke duration: 55.64 ± 27days). Intra-Class Correlation (ICC) and Bland Altman plot assessed intra-rater reliability and inter-rater reliability of CoP parameters during unstable sitting balance test (anteroposterior or mediolateral imbalance). Validity was established by correlating CoP parameters with the Modified Functional Reach Test, trunk strength, Balance Assessment in Sitting and Standing and Timed Up and Go tests.

**Results:**

The findings highlighted significant correlations between CoP parameters and trunk strength for anteroposterior seated destabilization. Good to excellent intra and inter-rater reliability (0.87 ≤ ICC ≤ 0.95) was observed for all CoP length parameters and CoP mean velocity in both mediolateral and anteroposterior imbalance conditions. CoP parameters for mediolateral unstable sitting condition were more reliable than for anteroposterior instability.

**Conclusion:**

Trunk control assessment during unstable sitting position on a seesaw is a reliable test for assessing trunk control ability in individuals after a stroke. CoP length and mean velocity are found to be the best parameters.

## Highlights

• Center of pressure values during anteroposterior perturbation is related to trunk strength.
• Center of pressure length parameters have excellent intra-rater and inter-rater reliability in individuals after a stroke in subacute phase.
• The reliability is better when the sitting instability is in mediolateral direction.

## 1 Introduction

Impaired postural control after a stroke is one of the main causes of limited functional recovery, limited independence and falls in persons with hemiparesis [1]. Thus, regaining postural control is one of the first goals in the rehabilitation process from subacute phase [2]. Asymmetry of weight distribution between the paretic and nonparetic sides as well as an increase in postural oscillations – particularly in the frontal plane – are systematically observed during sitting [3, 4], standing [1] and gait [5]. However, as the consequences of stroke are highly variable, it is necessary to test postural control with objective and reliable measures for an accurate description of postural control disorders.

After a stroke, assessment of sitting balance is crucial because trunk control impairments negatively influence standing balance [4], gait [6], upper limb functions [7, 8] and functional recovery [9]. Trunk control deficits seem to be associated with impairments of trunk proprioception [10] and trunk strength [11]. Two systematic reviews of trunk control assessment for individuals after stroke showed the dominance of the use of functional scales (e.g., Trunk Control Test) over computerized tools (e.g., stabilometric platform) [12, 13]. However, the correlation between scale scores and computerized assessment in sitting is highly variable [14]. This difference suggests that these tests do not assess the same capacities. Balance scales based on qualitative observation most often assess the ability to perform a task [12]. In contrast, quantitative tools such as force platform (FP) assess postural control using predictive, proactive, or reactive mechanisms [15]. FP tool seems very relevant in the context of individuals after stroke. Indeed, center of pressure (CoP) measures are reliable in standing position [16], also appear to be predictive of functional performance [5] and, as a biofeedback device, they make the exercises more effective [17].

Clinical assessment of balance should be done in static condition using a stable support, and dynamically using unstable support settings, and also during a functional task [15]. These three test modalities inform the clinician about different balance abilities that may be impaired following a stroke [18]. Static stability assesses the ability to stand unsupported by controlling center of mass on a stable support [15]. Dynamic stability assesses the ability to shift weight, controlling center of mass within the base of support. The functional tasks highlight balance capacities during other motor tasks, like reaching. In static sitting – compared to control persons – individuals with a history of a stroke show an increase of CoP length and velocity [3, 4, 19]. In dynamic sitting, Van Nes et al. 2008 observed an increase of CoP velocity after stroke when trunk postural control was tested on an air cushion [4]. Although disruptions were not dissociated according to the considered plane, results highlighted that instability was mostly visible for CoP parameters in the mediolateral direction – which was not observable in the static sitting test [4]. However, after a stroke, it is mainly trunk control in the frontal plane that is affected [20, 21], and CoP parameters during static sitting show greater instability in mediolateral than anteroposterior direction [4]. In addition, during a seated reaching task, reliability of the parameters is better for anteroposterior than mediolateral displacement [22, 23]. Thus, support-induced instability, allowing to dissociate the frontal and sagittal planes, seems relevant for individuals with hemiparesis. In sitting position, two studies used an unstable support generating a disturbance only in the frontal plane [20, 24]. The support being removable, it is easy to induce either mediolateral or anteroposterior disturbances, as already proposed for individuals with asymmetrical diseases such as scoliosis [25]. This unstable sitting test seems all the more important as a systematic review has shown the superiority of rehabilitation using an unstable surface rather than a stable surface [26]. Indeed, compared to static condition, dynamic condition has the advantage of increasing muscle activity to control center of mass, and of stimulating anticipatory postural adjustments. No previous studies on individuals with a history of a stroke has yet evaluated the measurement properties of CoP parameters for measuring trunk control ability during sitting tests with unstable supports. In healthy persons, CoP mean velocity reliability is excellent for this trunk control test [27].

The objective was to assess the validity and reliability of CoP parameters during an unstable sitting balance test on a seesaw for individuals after stroke in subacute phase. Our hypothesis was that CoP parameters correlate with trunk muscle strength. CoP length parameters were expected to demonstrate sufficient reliability to use this sitting test in clinical practice (ICC > 0.75) [28,29]. Nevertheless, CoP ellipse area may be less reliable [29]. As mediolateral instability is more disruptive than anteroposterior instability, CoP parameters are likely to demonstrate greater reliability for the frontal plane.

## 2 Materials and Methods

### 2.1 Study design

The chosen design was intra- and interrater reliability and validity study.

### 2.2 Participants

Based on an expected ICC ≥ 0.85, a significance level of 0.05 and power of 0.8, a sample size of 32 participants was required [30].

Inclusion criteria were the following: age 50 to 75, stroke > 1 week and < 3 months, medically stable, Mini-Mental State Examination (MMSE) ≥ 22 points [31] and individual able to sit during 30 s independently without upper limb support [32]. The choice of the early subacute phase (7 days – 3 months) was justified by the potential for motor recovery – which is highest during this phase – and the necessity to test trunk control early after stroke [33]. Individuals with additional trunk impairments such as idiopathic scoliosis and low back pain, pain, or medical complications were excluded.

Recruitment was conducted consecutively in the neurorehabilitation department of the Geneva University Hospitals. All participants gave their oral and written informed consent prior to data collection. This study was approved by the local ethics committee (Commission Cantonale d’Ethique de la Recherche sur l’être humain - CCER Geneva - 2018–02026).

### 2.3 Procedure

All tests were performed on a single day by two physiotherapists with at least two years of experience in neurology. During session 1, following the clinical tests – isometric trunk strength, Modified Functional Reach Test (MFRT), Balance Assessment in Sitting and Standing (BASSP) and Timed Up and Go test (TUG) –, two raters tested unstable sitting balance with a FP. After 2 – 4h of rest, rater 1 repeated the unstable sitting tests for session 2. Rater (1 or 2) and test (anteroposterior vs. mediolateral) orders were random. Since the FP is connected to a program which directly records data with no possible visualization, raters were blind to their own tests and to the other rater's.

### 2.4 Study outcomes

#### 2.4.1 Trunk isometric strength

To quantify trunk strength, a handheld dynamometer was used (MicroFET 2®, biometrics, Paris, France). After stroke, this measurement presents an excellent reliability [34]. Individuals were tested in sitting position. The dynamometer was successively and randomly placed over the lateral part of the trunk (under the axillary zone on the rib cage - paretic and nonparetic sides), the sternum (flexion) and the T4 vertebra (extension). Individuals had to exert an isometric maximum push against the dynamometer during 5 s. The maximum voluntary force value (MVF) was recorded (N). Two trials in each direction were performed with a 30 s rest between each trial.

#### 2.4.2 MFRT

The MFRT was conducted, using the reliable procedure for individuals after stroke described by Katz-Leurer et al. 2009 [23, 35]. The participant was in sitting position with hips, knees and ankles positioned at a 90°-flexion, feet placed on the floor. During the test, the participant did not touch the wall. Three movements were tested, each with three trials, as follows: 1) sitting with the unaffected side near the wall with the nonparetic upper limb in a 90°-flexion and leaning forward; 2) sitting back to the wall, leaning right and, 3) sitting back to the wall, leaning left. For lateral displacements, the anatomical landmark was the acromion so as not to put the upper limb at to 90° of abduction. The distance between the initial and final positions was recorded by marking the wrist or acromial location on the wall.

#### 2.4.3 BASSP

Standing and sitting balance were assessed using the BASSP tool based on 14 points, which shows good reliability and validity for individuals after stroke [36]. Postural reactions were assessed in standing and sitting positions during four rater-induced pushes towards the front, back, left and right, successively. A score of "0" – subject needs external support – to "4" – stable without external assistance – was given for after test. The BASSP test then assessed the subject's ability to pick up objects from the ground (front/left, front and front/right). A score of "0" – no ability to pick up objects – to "3" – objects picked up without external assistance – was given.

#### 2.4.4 TUG

Functional mobility was tested using the TUG, which has excellent reliability in post-stroke context [37]. From a seated position, the participant was instructed to stand up, walk 3 m, turn around a cone, and then walk 3 m back to sit back down. The time (s) needed to complete the task was recorded. Each subject did one trial.

#### 2.4.5 Dynamic sitting balance on a seesaw

**Figure 1:**
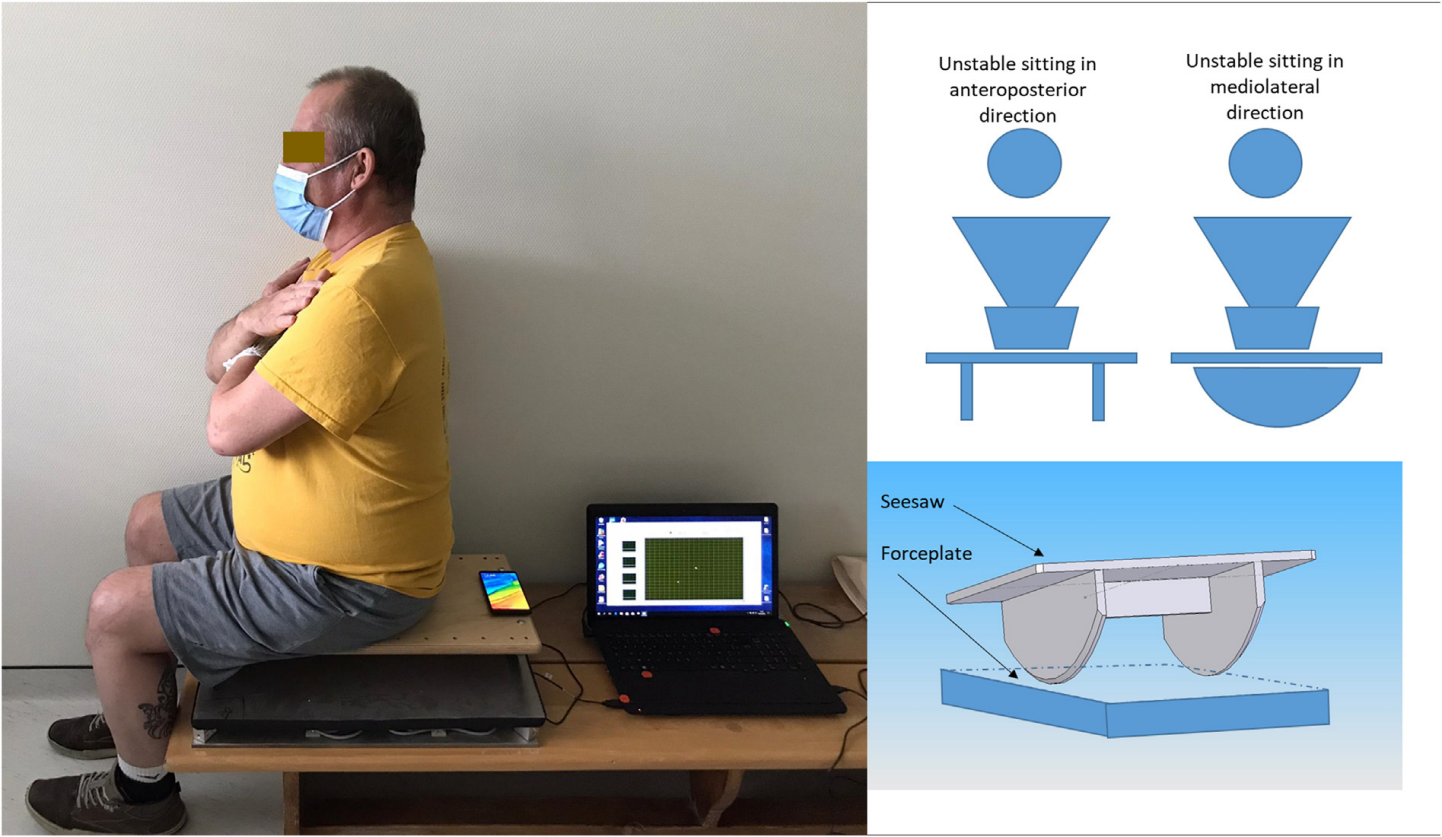
Experimental set-up for the mediolateral unstable sitting test on a seesaw.

The testing device consisted of a wooden bench, a FP (kinétools 2015, Kicarre company) and a seesaw (60 cm × 35 cm with a height of 9.5cm, Balance board 60 Pedalo®) (Figure 1) [24,25]. The FP's dimensions were 60 cm × 45 cm x 6.2 cm; it consisted of two plates with four force transducers (SP4C3-MR, precision C3, HBM®) connected to a converter (NI USB-6009 DAQ, 14/8 inputs resolution, 48 ks/s) allowing rapid integration to a signal processing software (LabVIEW 2016). Before processing the signals, they were filtered with a 5^th^ Order Butterworth Low Pass Filter (cut-off set to 45 Hz).

Subject was seated on the seesaw, feet flat on the floor with knees and hips in flexion. For each test, participants were instructed to be as stable as possible with their eyes open with both arms crossed over their torso. Prior to the test, the rater verified that the participant was not leaning with their legs on the bench. The position of the seesaw was used to induce a disturbance either in the mediolateral or in the anteroposterior direction. A smartphone using Clinometer was placed on the seesaw to check horizontality at the beginning of the test. The FP recorded CoP evolution during 10 s at a sampling frequency of 100 Hz [29]. Two trials were performed for each disturbance setting. During the test, the rater was at the participant's side to secure them and prevent falls.

### 2.5 Data treatment and statistics

Data was processed with Python (v.3.8.3) using the SciPy package (v.1.5.2). This package is open source - OSI (Open Source Initiative) - approved and modified by the BSD (Berkeley Software Distribution) license (3-clause). SPSS software (IBM statistics software v.20.0 - Armonk, New York, USA) was used for statistics.

Trunk MVF was normalized with the subject's weight according to the following formula: MVF_normalized_ = [MVF (N) / Weight (kg)] ∗ 100.

MFRT values were normalized with subject's height: MFRT_normalized_ = [Distance (cm) / Height (cm)] ∗ 100.

For each unstable sitting test (anteroposterior and mediolateral), CoP variables were extracted: length (total, anteroposterior and mediolateral), ellipse area (CI95%), deltas (the range between maximal and minimal values anteroposterior and mediolateral), mean and maximum velocity as well as variability. Variability was the standard deviation (SD) of the CoP displacement during the 10 s of the test. For each parameter and test, the two trials were averaged for each disturbance setting.

For descriptive statistics, frequencies or means and SD were calculated. For concurrent validity, after testing data normality with the Shapiro-Wilk test, Pearson correlation coefficients (r) were calculated between each CoP parameter and the values of the MVF_normalized_ (anterior, posterior, paretic, nonparetic), the MFRT_normalized_ (anterior, paretic, nonparetic), the BASSP and TUG tests. Results were considered statistically significant when *p* was < 0.05.

Intra-rater reliability was assessed using the ICC_(3,k)_ model and inter-rater reliability was assessed using the ICC_(2,k)_ model [38]. The values obtained were interpreted according to the following thresholds: ICC less than 0.50 = poor reliability, ICC between 0.50 and 0.75 = moderate, ICC between 0.75 and 0.90 = good and an ICC greater than 0.90 was considered excellent [38]. If ICC was greater than or equal to 0.75, the standard error of measurement (SEM) was calculated [39]: SEM = SD ∗ [√ (1 - ICC)]

The SD was the SD of all the measurements in the session 1 and session 2.

The minimum detectable change (MDC) was calculated as follows:

MDC=SEM∗1.96∗√2

Absolute reliability was investigated using the Bland-Altman analysis to determine between-session or between-rater agreement. The 95 % limits of agreement (LOA_95%_) represent 1.96 SD above and below the mean difference (bias) between sessions.

## 3 Results

Thirty-two individuals after stroke with mean age: 64.34 ± 9.30 years were included in this study. Demographic characteristics and clinical tests are presented in Table 1.

**Table 1:** Demographic and clinical characteristics of included participants.

Variables	N = 32
Age (years)	64.34 ± 9.30
Height (m)	1.73 ± 0.09
Weight (kg)	75.17 ± 13.87
Body Mass Index (kg/m^2^)	24.98 ± 3.28
Gender	9 women / 23 men
Stroke type	12 hemorrhagic / 20 ischemic
Post-stroke duration (days)	55.64 ± 26.56 [Min: 21; Max: 86]
Hemiparesis side	10 right / 22 left
Mini-Mental State Examination (score/30)	25.62 ± 2.67
Balance assessment in sitting and standing position (BASSP) (/14 points)	12.20 ± 3.15
Trunk strength (MVF_normalized_ - %) Paretic side Nonparetic side Anterior Posterior	32.29 ± 11.21 32.11 ± 8.31 36.50 ± 11.77 47.81 ± 12.09
Modified Functional Reach Test (MFRT_normalized_ - %) Anterior Paretic side Nonparetic side	16.63 ± 5.21 10.22 ± 2.96 10.14 ± 2.69
Timed Up and Go test (s)	17.86 ± 14.78

MVF = Maximal voluntary force, MFRT = Modified Functional Reach Test.

### 3.1 Concurrent validity

For *the unstable sitting test in the anteroposterior direction*, CoP total length was significantly correlated with forward displacement of the MFRT_normalized_ (r = -0.36, *p = 0.046*) as well as with the MVF_normalized_ on the paretic (r = -0.41, p = 0.021) and posterior (r = -0.41, *p = 0.021*) sides (Table 2).

**Table 2:** Validity results for all CoP parameters. The coefficient correlation (r) is reported with p value in brackets.

		MFRT			BASSP	Muscular strength				TUG
		Anterior	Paretic side	Nonparetic side		Paretic side	Nonparetic side	Anterior	Posterior	
Anteroposterior unstable sitting test	Total path length	***-0.36 (0.046)***	-0.02 (NS)	-0.23 (NS)	0.05 (NS)	***-0.41 (0.021)***	-0.32 (NS)	-0.24 (NS)	***-0.41 (0.021)***	0.17 (NS)
	Mean velocity	***-0.36 (0.046)***	-0.02 (NS)	-0.23 (NS)	0.09 (NS)	***-0.41 (0.021)***	-0.32 (NS)	-0.24 (NS)	***-0.41 (0.021)***	0.17 (NS)
	AP length	-0.33 (NS)	-0.03 (NS)	-0.20 (NS)	0.04 (NS)	***-0.44 (0.014)***	***-0.39 (0.030)***	-0.26 (NS)	***-0.40 (0.026)***	0.10 (NS)
	ML length	***-0.35 (0.050)***	-0.11 (NS)	-0.25 (NS)	0.06 (NS)	-0.29 (NS)	-0.13 (NS)	-0.14 (NS)	***-0.37 (0.041)***	0.21 (NS)
Mediolateral unstable sitting test	Total path length	-0.32 (NS)	-0.02 (NS)	-0.15 (NS)	0.14 (NS)	-0.33 (NS)	-0.34 (NS)	-0.20 (NS)	-0.26 (NS)	-0.09 (NS)
	Mean velocity	-0.32 (NS)	-0.02 (NS)	-0.15 (NS)	0.21 (NS)	-0.33 (NS)	-0.34 (NS)	-0.20 (NS)	-0.26 (NS)	-0.09 (NS)
	ML length	-0.30 (NS)	-0.05 (NS)	-0.15 (NS)	0.13 (NS)	-0.32 (NS)	***-0.39 (0.035)***	-0.23 (NS)	-0.29 (NS)	-0.04 (NS)
	AP length	-0.34 (NS)	-0.09 (NS)	-0.12 (NS)	0.17 (NS)	-0.35 (NS)	-0.34 (NS)	-0.16 (NS)	-0.22 (NS)	-0.16 (NS)

NS = Non-significant result, MFRT = Modified Functional Reach Test, BASSP = Balance Assessment in Sitting and Standing, TUG = Timed Up and Go test.

Anteroposterior CoP length values significantly correlated with the MVF_normalized_ on the paretic (r = -0.44, *p = 0.014*), nonparetic (r = -0.39, *p = 0.030*) and posterior (r = -0.40, *p = 0.026*) values. Mediolateral CoP length values correlated with the MVF_normalized_ for posterior test (r = -0.37, *p = 0.041*). No significant correlations were found between CoP parameters and the BASSP and TUG clinical tests.

For *the unstable sitting test in the mediolateral direction*, only mediolateral CoP length correlated with MVF_normalized_ on the nonparetic side (r = -0.39, *p = 0.035*). All other parameters were non-significant.

### 3.2 Intra-rater reliability

The ICC, SEM and MDC for intra- and inter-rater reliability are presented in Table 3 for the anteroposterior unstable sitting test and in Table 4 for the mediolateral unstable sitting test.

**Table 3:** Inter and intra-rater reliability for CoP parameters during anteroposterior unstable sitting test.

Parameters	Session 1		Session 2	Inter-rater reliability		Intra-rater reliability	
	Rater 1 (mean ± standard deviation)	Rater 2 (mean ± standard deviation)	Rater 1 (mean ± standard deviation)	ICC 2,k [CI95%]	SEM; MDC	ICC 3,k [CI95%]	SEM; MDC
Total path length (mm)	267.70 ± 49.72	273.81 ± 60.20	273.41 ± 57.11	**0.92 [0.85–0.96]**	14.51; 40.09	0.92 [0.83–0.96]	15.20; 41.99
Area (mm^2^)	9.94 ± 12.67	8.33 ± 11.75	11.06 ± 15.87	0.18 [-0.07 – 0.61]	NA	0.05 [-1.10 – 0.56]	NA
Max velocity (mm/s)	90.38 ± 19.05	97.09 ± 32.33	97.07 ± 35.07	0.51 [0.00–0.76]	NA	0.31 [-0.43 – 0.68]	NA
Mean velocity (mm/s)	26.77 ± 4.97	27.38 ± 6.02	27.34 ± 5.71	**0.92 [0.85–0.96]**	1.55; 4.28	**0.92 [0.83–0.96]**	1.52; 4.19
AP length (mm)	202.13 ± 39.82	206.00 ± 48.38	206.67 ± 42.63	**0.90 [0.79–0.95]**	13.91; 38.44	**0.88 [0.76–0.95**	14.35; 36.65
AP delta (mm)	4.12 ± 1.72	4.14 ± 2.16	4.70 ± 2.34	**0.76 [0.50–0.89]**	0.95; 2.63	0.59 [0.15–0.81]	NA
AP variability (mm)	0.85 ± 1.14	0.91 ± 1.14	1.43 ± 2.25	0.64 [0.25–0.83]	NA	0.37 [0.30–0.70]	NA
ML length (mm)	135.10 ± 27.05	139.38 ± 29.52	137.99 ± 30.15	**0.95 [0.89–0.98]**	6.29; 17.40	**0.93 [0.85–0.97]**	7.62; 21.05
ML delta (mm)	3.26 ± 2.84	2.54 ± 1.57	2.77 ± 1.45	0.00 [-1.03 – 0.51]	NA	0.16 [-0.79 – 0.60]	NA
ML variability (mm)	1.07 ± 2.82	0.53 ± 1.00	0.57 ± 0.89	0.08 [-124 – 0.48]	NA	0.04 [-1.22 – 0.51]	NA

ML = mediolateral, AP = anteroposterieur, NA = non applicable, ICC = Intraclass correlation coefficient, SEM = Standard Error of Measurement, MDC = Minimal Detectable Change, CI = Confidence Interval.

**Table 4:** Inter and intra-rater reliability for CoP parameters during mediolateral unstable sitting test.

Parameters	Session 1		Session 2	Inter-rater reliability		Intra-rater reliability	
	Rater 1 (mean ± standard deviation)	Rater 2 (mean ± standard deviation)	Rater 1 (mean ± standard deviation)	ICC 2,k [CI95%]	SEM; MDC	ICC 3,k [CI95%]	SEM; MDC
Total path length (mm)	278.99 ± 50.76	276.29 ± 47.17	278.84 ± 53.84	**0.92 [0.84–0.96]**	13.74; 37.97	**0.92 [0.84–0.97]**	14.72; 40.68
Area (mm^2^)	23.47 ± 25.56	16.84 ± 16.48	19.38 ± 30.21	**0.80 [0.57–0.91]**	9.64; 26.66	**0.79 [0.55–0.90]**	12.77; 35.29
Max velocity (mm/s)	98.09 ± 30.34	93.76 ± 18.43	97.97 ± 22.61	0.74 [0.46–0.88]	NA	0.73 [0.40–0.87]	NA
Mean velocity (mm/s)	27.90 ± 5.08	27.63 ± 4.72	27.88 ± 5.38	**0.92 [0.84–0.97]**	1.28; 3.55	**0.92 [0.84–0.97]**	1.47; 4.06
AP length (mm)	214.49 ± 39.84	210.27 ± 37.76	214.17 ± 45.89	**0.87 [0.72–0.94]**	13.89; 38.39	**0.91 [0.80–0.96]**	12.82; 35.43
AP delta (mm)	5.56 ± 3.53	4.70 ± 2.26	5.07 ± 4.39	0.68 [0.33–0.85]	NA	**0.88 [0.74–0.94]**	1.38; 3.80
AP variability (mm)	2.83 ± 7.58	1.12 ± 1.39	2.04 ± 6.41	0.12 [0.08–0.58]	NA	**0.98 [0.95–0.99]**	1.00; 2.75
ML length (mm)	137.05 ± 25.83	137.87 ± 23.66	136.83 ± 23.81	**0.95 [0.90–0.98]**	5.49; 15.17	**0.89 [0.77–0.95]**	8.20; 22.67
ML delta (mm)	5.21 ± 2.45	5.14 ± 1.84	4.70 ± 1.89	**0.78 [0.54–0.90]**	1.00; 2.79	0.70 [0.37–0.86]	NA
ML variability (mm)	1.91 ± 2.30	1.55 ± 1.17	1.38 ± 1.29	0.69 [0.35–0.86]	NA	0.51 [-0.03 – 0.77]	NA

ML = mediolateral, AP = anteroposterieur, NA = non applicable, ICC = Intraclass correlation coefficient, SEM = Standard Error of Measurement, MDC = Minimal Detectable Change, CI = Confidence Interval.

**Figure 2:**
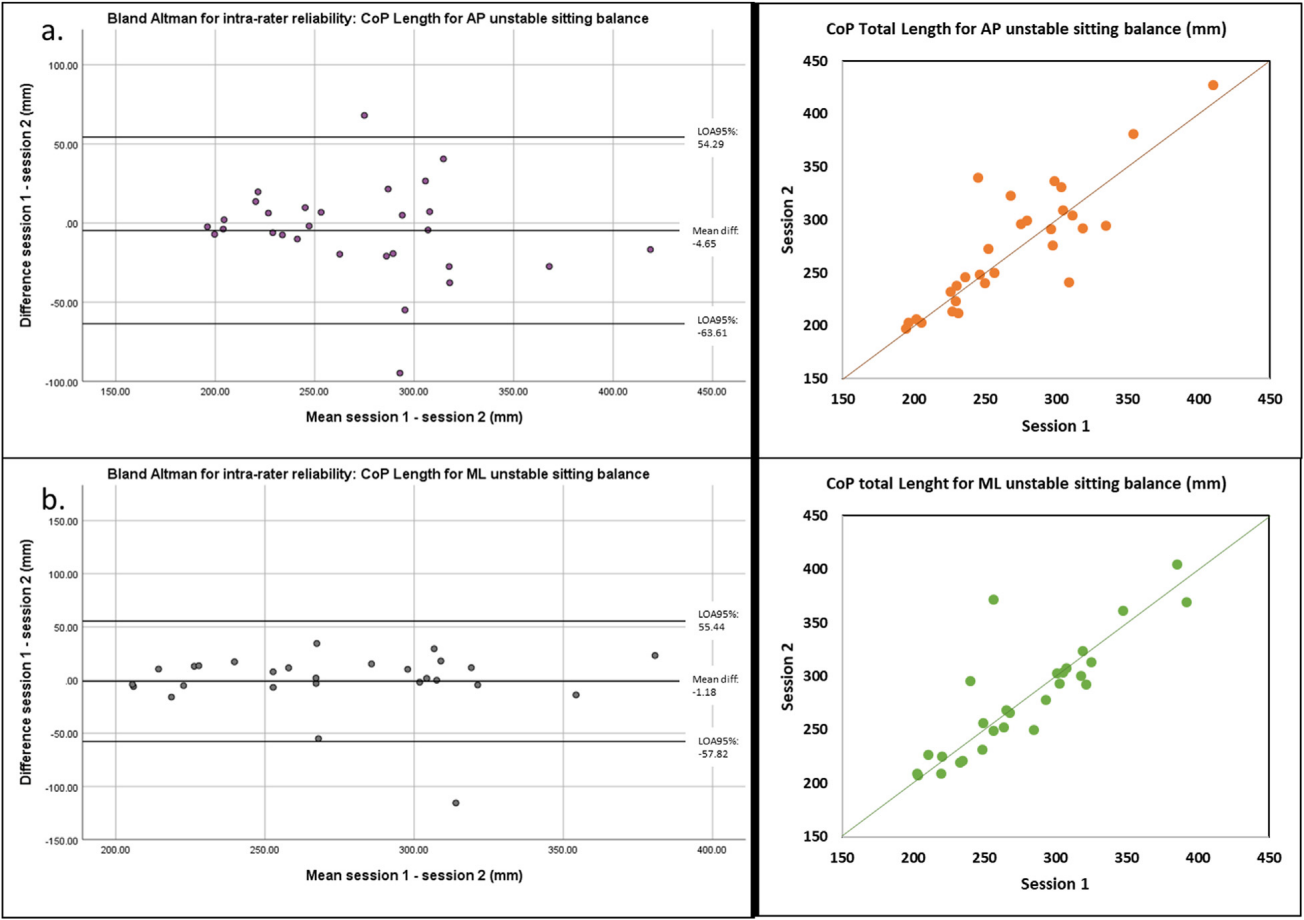
Left: Bland and Altman plots for intra-rater reliability for anteroposterior (AP – 2.a) and mediolateral (ML – 2.b) unstable sitting balance tests. Right: scatter plots for anteroposterior (AP, in orange) and mediolateral (ML, in green).

When the seesaw induced an anteroposterior destabilization, intra-rater reliability was excellent for total CoP length (ICC_(3,k)_ = 0.92, Table 3) with a bias between session 1 and 2 of -4.65 mm (LOA95% = -63.61; 54.29) (Figure 2.a.). Good-to-excellent reliability was observed for mean velocity, anteroposterior and mediolateral CoP lengths (ICC_(3,k)_ ≥ 0.88). Nevertheless, the intra-rater ICC_(3,k)_ values were poor-to-moderate for CoP ellipse area, maximum velocity, deltas, and variabilities.

Intra-rater reliability for the mediolateral unstable sitting highlighted excellent reliability for total CoP length (ICC_(3,k)_ = 0.92) with a bias inter-session error of -1.18 mm (LOA95% = -57.82; 55.44) (Figure 2.b.).

For this same mediolateral test, good-to-excellent reliability was observed for mediolateral and anteroposterior CoP lengths, ellipse area, anteroposterior delta and anteroposterior variability (0.79 ≤ ICC_(3,k)_ ≤ 0.98), while reliability was moderate for maximum velocity and mediolateral delta.

### 3.3 Inter-rater reliability

**Figure 3:**
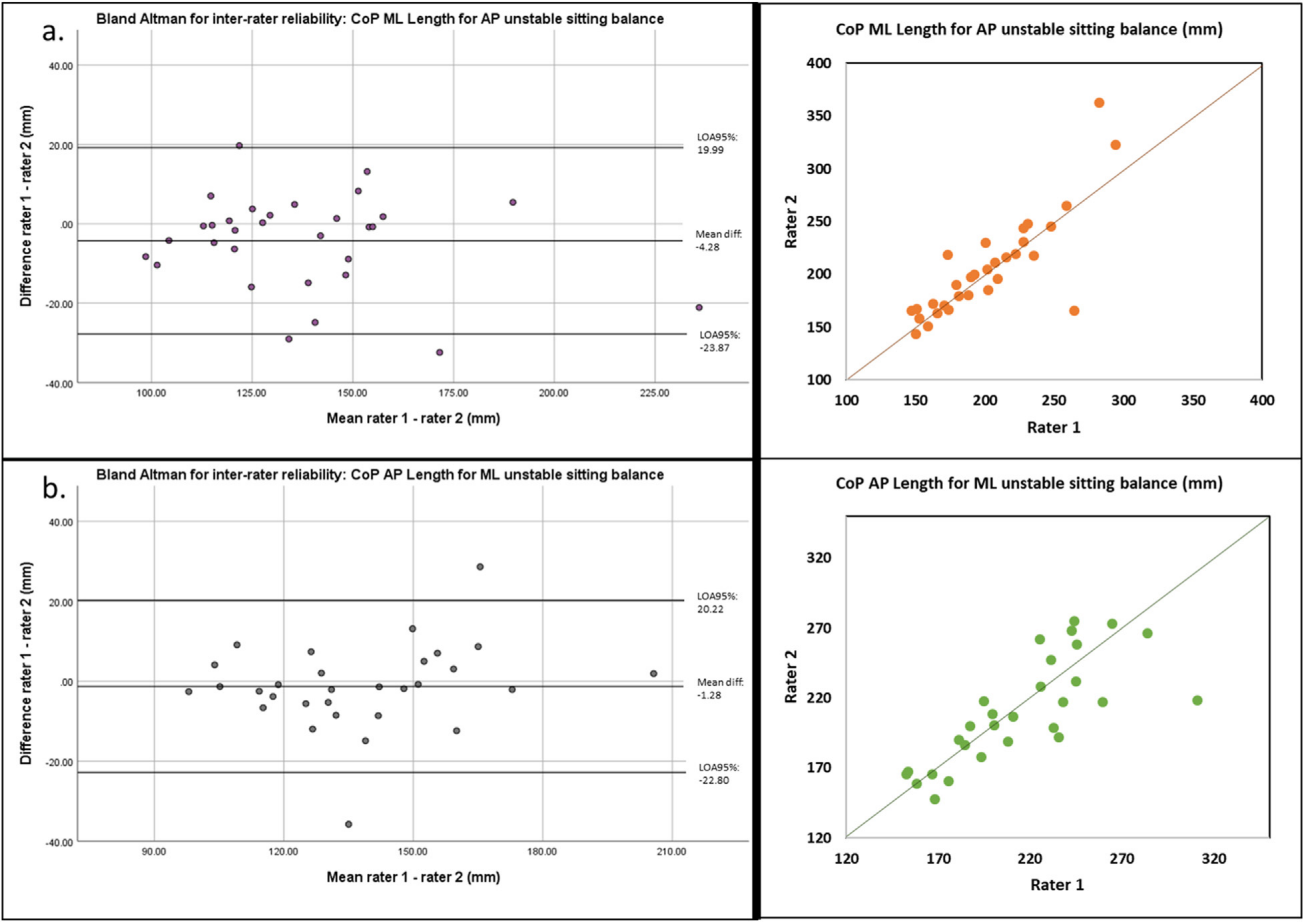
Left: Bland and Altman plots for inter-rater reliability for anteroposterior (AP – 3.a) and mediolateral (ML – 3.b) unstable sitting balance tests. In AP direction, the CoP ML length is presented and in ML direction, the CoP AP length is presented. Right: scatter plots for anterosterior (AP, in orange) and mediolateral (ML, in green).

For the anteroposterior unstable sitting test, inter-rater reliability was excellent for all CoP length parameters and mean velocity (0.90 ≤ ICC_(2,k)_ ≤ 0.95) (Table 3). For the mediolateral CoP length, the bias between raters 1 and 2 was -4.28 mm (LOA95% = -23.47; 19.99) (Figure 3.a.). ICC_(2,k)_ was good for the anteroposterior delta (ICC_(2,k)_ = 0.76), whereas inter-rater reliability was poor-to-moderate for CoP ellipse area, maximum velocity, mediolateral delta and variabilities (0.00 ≤ ICC_(2,k)_ ≤ 0.64).

The unstable sitting test in the mediolateral direction was characterized by a good-to-excellent inter-rater reliability for all CoP length parameters, mean velocity, ellipse area and mediolateral delta (0.78 ≤ ICC_(2,k)_ ≤ 0.95, Table 4). Bias between raters was -1.28mm (LOA95% = -22.80; 20.22) for the anteroposterior CoP length parameter (Figure 3.b.). Moderate inter-rater reliability was observed for maximum velocity, anteroposterior delta and mediolateral variability (0.66 ≤ ICC_(2,k)_ ≤ 0.74). Only ICC_(2,k)_ was poor for anteroposterior CoP variability.

## 4 Discussion

This study assessed, for individuals with hemiparesis after a stroke in subacute phase, the validity and reliability of CoP parameters measured during unstable sitting on a seesaw. CoP parameters were mainly associated with the MVF_normalized_ isometric trunk test. The reliability analysis highlighted an excellent intra- and inter-rater reliability of CoP length parameters and better ICC when the disturbance was in the frontal plane. CoP ellipse area, deltas and variability do not appear to be reliable enough for use in clinical practice.

### 4.1 Concurrent validity

Our results confirm the link between trunk control ability and trunk muscle deficits. Karthikbabu et al. 2021 [11] found higher correlations between the MVF and Trunk Impairment Scale (TIS) performance. This difference could be explained by stroke stages (chronic or subacute), the variable analyzed (TIS score vs. CoP value), and the task performed. Indeed, the TIS assesses trunk control during a functional task while the unstable sitting test assesses the ability to maintain a stable sitting position despite the disturbance generated by the seesaw. For the TIS, the direction of the disturbance is predictable, while the seesaw's unstable support induces an unpredictable direction of the disturbance as the destabilization can be either anterior or posterior [40]. Thus, both these tests assess the anticipated postural adjustments but with distinct components, and confirm the complementarity of the dynamic and functional tests in sitting position [15]. The moderate and non-systematic correlations between CoP values and MFRT_normalized_ performance also support the specificity of the unstable sitting balance test to the reaching tasks as previously demonstrated in standing [41]. When correlations are less than 0.4, either the test is not reliable, or both tests evaluate different motor abilities [22]. Given the excellent reliability obtained, we will retain the second interpretation. Thus, the absence of correlation with TUG and BASSP tests shows the interest of a specific analysis of trunk control with CoP measures in an unstable sitting position.

### 4.2 Reliability

Excellent intra-rater reliability was observed for CoP length parameters, confirming the results obtained on the reaching task in individuals after stroke [22]. As Barbado et al. 2017 did for a sitting task with unstable support in healthy persons [27], we observed an excellent reliability for the CoP mean velocity parameter. During the dynamic sitting test, when the subject's center of mass moves, the seesaw rolls, thus inducing a progressive acceleration in a direction that requires rapid postural readjustments to avoid falling [6]. Therefore, the mean velocity and CoP length – reflecting the energy required to maintain balance – are parameters that seem particularly suitable and reliable to describe postural readjustments during external instability induced around the pelvis. However, as has been observed in stable and unstable standing for individuals after stroke [42, 43] and healthy controls [29], the CoP ellipse area is less reliable.

The inter-rater reliability was also excellent for CoP length parameters and mean velocity, demonstrating that the unstable sitting test can be used in clinical practice in stroke context, even by different raters.

The originality of this study was to test reliability by differentiating the instability planes (frontal vs. sagittal). Indeed, the seesaw can either generate instability in the frontal plane or in the sagittal plane separately. This approach allows a fine analysis of the postural readjustments produced in the plane perpendicular to the disturbance induced by the seesaw [25]. The results showed close ICC values between anteroposterior and mediolateral tests for the CoP total length. Nevertheless, a better reliability of the other CoP parameters was observed when the destabilization was in the mediolateral direction, which could be related to the consequences of stroke. Indeed, a previous study highlighted that during unstable sitting in the frontal plane, post-stroke individuals had the ability to self-regulate trunk control impacted by the distortion of internal systems involved in mediolateral balance regulation [20]. Moreover, trunk control is particularly affected in the frontal plane when walking, due to asymmetries and increased accelerations towards the paretic side [21]. Thus, the consequences of stroke would particularly affect postural readjustments in the frontal plane with CoP parameters more variable, which could explain better reliability when compared to sagittal unstable sitting.

### 4.3 Clinical implication

Considering the many factors involved in balance [44], no simple test allows to assess it in all its components. Thus, it is necessary to test static sitting balance (stable support), dynamic sitting balance (unstable support) and sitting balance during a functional task (reaching) [15]. Sitting is often assessed through a reaching task, which is a trunk self-destabilization in a predictable direction originating from the shoulders [22, 23]. The seesaw test induces a trunk destabilization that originates from the pelvis in an unpredictable direction, which seems particularly adapted for patients with pelvis instability during gait [6]. The moderate correlations between the MFRT_normalized_ and sitting balance tests tend to show that these tests provide complementary information for the treating of patients after stroke. These tests are essential for proposing effective trunk control exercises in the subacute stroke phase. Indeed, this aspect is still too often neglected, while the benefits are important for postural control in sitting, standing and gait [6, 21].

The FP tool is often considered relevant, but the cost and complexity of the analysis can be a hurdle to its use [16]. However, more and more neurorehabilitation centers have these tools that allow for very precise evaluation of postural adjustments. Previously, Näf et al. 2020 [22] highlighted the reliability of CoP values for a stable sitting test and during reaching task after a stroke. Therefore, the CoP length (total, anteroposterior and mediolateral) and CoP Mean velocity are reliable FP parameters for assessing static, dynamic and functional tasks in sitting position, as well as postural control while standing [16]. Care should be taken with the use of ellipse area, which is often a parameter used in clinical practice. The FP can be used in various balance conditions, and for patients with different diseases. In addition, this tool can provide feedback during balance exercises, improving their effectiveness [17]. Therefore, the clinical applications of the FP are very broad, both for patient assessment and for feedback during rehabilitation. When clinicians do not have a FP, the Function In Sitting test could be an interesting alternative because it measures proactive and reactive adjustments as well as static sitting balance [45].

### 4.4 Study limitation

This study's main limitation results from the selection criteria of individuals after stroke. To ensure the participants' ability to perform the tests, we chose inclusion criteria to ensure feasibility. However, the results of the tests show mild-to-moderate motor impairment which limits the transfer of results for patients with more severe motor deficits. No adverse events occurred during the entire procedure for all included participants. Only one subject was unable to complete the entire test due to fatigue. Results therefore target patients with moderate impairments and do not apply to the most affected patients.

## 5 Conclusion

CoP measurements during the unstable seated balance test on a seesaw in the frontal and sagittal planes appear to be valid parameters for assessing patients after a stroke in the subacute phase. However, reliability was higher for CoP length and velocity compared to ellipse area, delta, or variability parameters. While performance was associated with trunk MVF_normalized_ and the MFRT_normalized_, no correlation was found with the BASSP and TUG tests. Future studies should evaluate a control group to better understand the impact of brain injury on postural readjustments during unstable sitting balance. Finally, the predictive value of this test for functional recovery should be assessed.

## Declarations

### Author contribution statement

Anne-Violette Bruyneel: Conceived and designed the experiments; Performed the experiments; Analyzed and interpreted the data; Contributed reagents, materials, analysis tools or data; Wrote the paper.

Serge Mesure; Emmanuel Guyen: Conceived and designed the experiments; Wrote the paper.

Aline Reinmann: Performed the experiments; Analyzed and interpreted the data; Contributed reagents, materials, analysis tools or data.

Caroline Sordet; Pablo Venturelli; Irmgard Feldmann: Performed the experiments.

### Funding statement

Professor (associate) Anne-Violette Bruyneel was supported by HES-SO University of Applied Sciences and Arts of WesternSwitzerland [FRI grant 02-A18]. The sponsor had no role in the conception, design, methods, data analysis, and preparation of this article.

### Data availability statement

Data will be made available on request.

### Declaration of interest's statement

The authors declare no conflict of interest.

### Additional information

The clinical trial described in this paper was registered at NCT under the registration number NCT04639453.
